# Screening and Functional Validation of the Chlorophyll Synthesis-Related Gene *StaHemF* in *Sinobambusa tootsik* f. *albostriata*

**DOI:** 10.3390/ijms27167186

**Published:** 2026-08-11

**Authors:** Xinru Gao, Zonghui Wei, Yuhan Lin, Jundong Rong, Tianyou He, Yushan Zheng, Shuming Liu, Lingyan Chen

**Affiliations:** 1 College of Forestry, Fujian Agriculture and Forestry University, Fuzhou 350002, China; gxr15529009057@163.com (X.G.); rongjd@126.com (J.R.); zys1960@163.com (Y.Z.); 2 College of Landscape Architecture and Art, Fujian Agriculture and Forestry University, Fuzhou 350002, China; 15161883229@163.com (Z.W.); 18057445945@163.com (Y.L.); hetianyou1985@163.com (T.H.); 3 Fuzhou Sanjiangkou Botanical Garden, Fuzhou 350000, China; fzssjklsm@126.com

**Keywords:** *Sinobambusa tootsik* f. *albostriata*, leaf color variation, *StaHemF*, transcriptome, chlorophyll accumulation

## Abstract

Leaf color variation is an important ornamental trait in bamboo and is primarily determined by chlorophyll accumulation. However, the molecular mechanisms underlying leaf color differentiation in *Sinobambusa tootsik* f. *albostriata* remain largely unclear. In this study, fully green (WG) and fully white (WW) leaf buds at three developmental stages (S1–S3) were analyzed to investigate the regulatory mechanisms associated with chlorophyll accumulation and identify key functional genes. Chlorophyll content determination, coproporphyrinogen III oxidase (CPOX) activity assays, and comparative transcriptome analysis were integrated to identify candidate genes involved in leaf color formation. Six *StaHemF* family members were identified, and *StaHemF5* was selected as the primary candidate based on phylogenetic relationships and expression patterns. Compared with WG leaf buds, WW leaf buds exhibited significantly reduced chlorophyll contents and CPOX activities throughout development, indicating impaired chlorophyll biosynthesis. *StaHemF5* encodes a chloroplast-localized protein and displays consistent expression trends between transcriptome analysis and qRT-PCR validation. Functional characterization revealed that transient overexpression of *StaHemF5* in *Nicotiana benthamiana* significantly enhanced CPOX activity and chlorophyll accumulation, suggesting a positive role of *StaHemF5* in chlorophyll biosynthesis. Collectively, these results demonstrate that StaHemF5 is a conserved HemF family member involved in chlorophyll accumulation and contributes to leaf color differentiation in *S. tootsik* f. *albostriata*. This study provides new insights into the molecular mechanisms underlying bamboo leaf color variation and identifies a potential candidate gene for the genetic improvement of ornamental bamboo.

## 1. Introduction

Leaf color variation represents an important ornamental trait in bamboo and provides valuable genetic resources for investigating the regulatory mechanisms of chloroplast development and chlorophyll metabolism [1]. Chlorophyll content and accumulation are major determinants of leaf coloration and photosynthetic capacity in plants, whereas defects in chlorophyll biosynthesis or metabolism often result in leaf color mutants, including albino, chlorotic, and variegated phenotypes [2,3]. During long-term evolution and domestication, bamboo species have accumulated abundant variegated germplasm resources, offering excellent materials for dissecting the molecular mechanisms underlying leaf color formation. *Sinobambusa tootsik* f. *albostriata* is a variegated ornamental bamboo characterized by stable green–white mosaic leaf coloration [4]. This species exhibits diverse leaf color phenotypes, including fully green (WG) and fully white (WW) leaves. Previous studies on *S. tootsik* f. *albostriata* have primarily focused on leaf anatomical characteristics and photosynthetic physiological responses [5,6]. However, the molecular mechanisms governing leaf color formation in this species remain largely unknown and have not been systematically investigated [7].

Chlorophyll accumulation is a complex enzyme-mediated process that occurs in chloroplasts, and alterations in the activities of key enzymes can significantly influence chlorophyll biosynthesis and chloroplast development [8,9,10]. Coproporphyrinogen III oxidase (CPOX) is a key enzyme in the chlorophyll biosynthetic pathway and is encoded by the HemF gene. It catalyzes the oxidative conversion of coproporphyrinogen III to protoporphyrin IX, a critical intermediate in tetrapyrrole biosynthesis [11]. Previous studies have demonstrated that defects in HemF function can impair chlorophyll accumulation, disrupt chloroplast development, and result in albino or chlorotic phenotypes [12]. In maize, cyanobacteria, and soybean, HemF homologs have been shown to participate in tetrapyrrole metabolism, chlorophyll biosynthesis, and chloroplast development [12,13,14]. However, the evolutionary characteristics and functional roles of the HemF gene family in bamboo species remain largely unexplored. In particular, whether specific HemF members contribute to the formation of green–white variegation in *S. tootsik* f. *albostriata* remains unknown.

To elucidate the molecular mechanisms underlying leaf color variation in *S. tootsik* f. *albostriata*, leaf buds exhibiting distinct color phenotypes were selected as experimental materials in this study. Chlorophyll content and CPOX enzyme activity were initially determined to evaluate differences in chlorophyll biosynthetic capacity among different leaf color phenotypes. Subsequently, transcriptome data were integrated to identify *StaHemF* family members, and candidate functional genes were further screened based on phylogenetic relationships and expression patterns. Finally, the biological function of the key candidate gene *StaHemF5* was evaluated using a transient expression system in *Nicotiana benthamiana*. This study provides insights into the potential role of *StaHemF5* in chlorophyll accumulation and leaf color formation in *S. tootsik* f. *albostriata*. Furthermore, these findings provide valuable genetic resources and a theoretical foundation for understanding and improving leaf color traits in ornamental bamboo.

## 2. Results

### 2.1. Transcriptomic Analysis and Differentially Expressed Gene (DEG) Identification

After removing low-quality reads, all samples generated more than 6 Gb of clean data. Three biological replicates were prepared for each phenotype at each developmental stage. The sequencing error rate remained consistently low (0.01%) across all samples. The Q20 values ranged from 98.44% to 98.58%, and all Q30 values exceeded 95.34%. The GC content ranged from 53.43% to 53.73% in WW leaf buds and from 54.23% to 54.82% in WG leaf buds (Table S1). These quality control results demonstrated that the transcriptome sequencing data exhibited high accuracy and reliability, providing a solid foundation for subsequent transcriptomic analyses.

**Figure 1:**
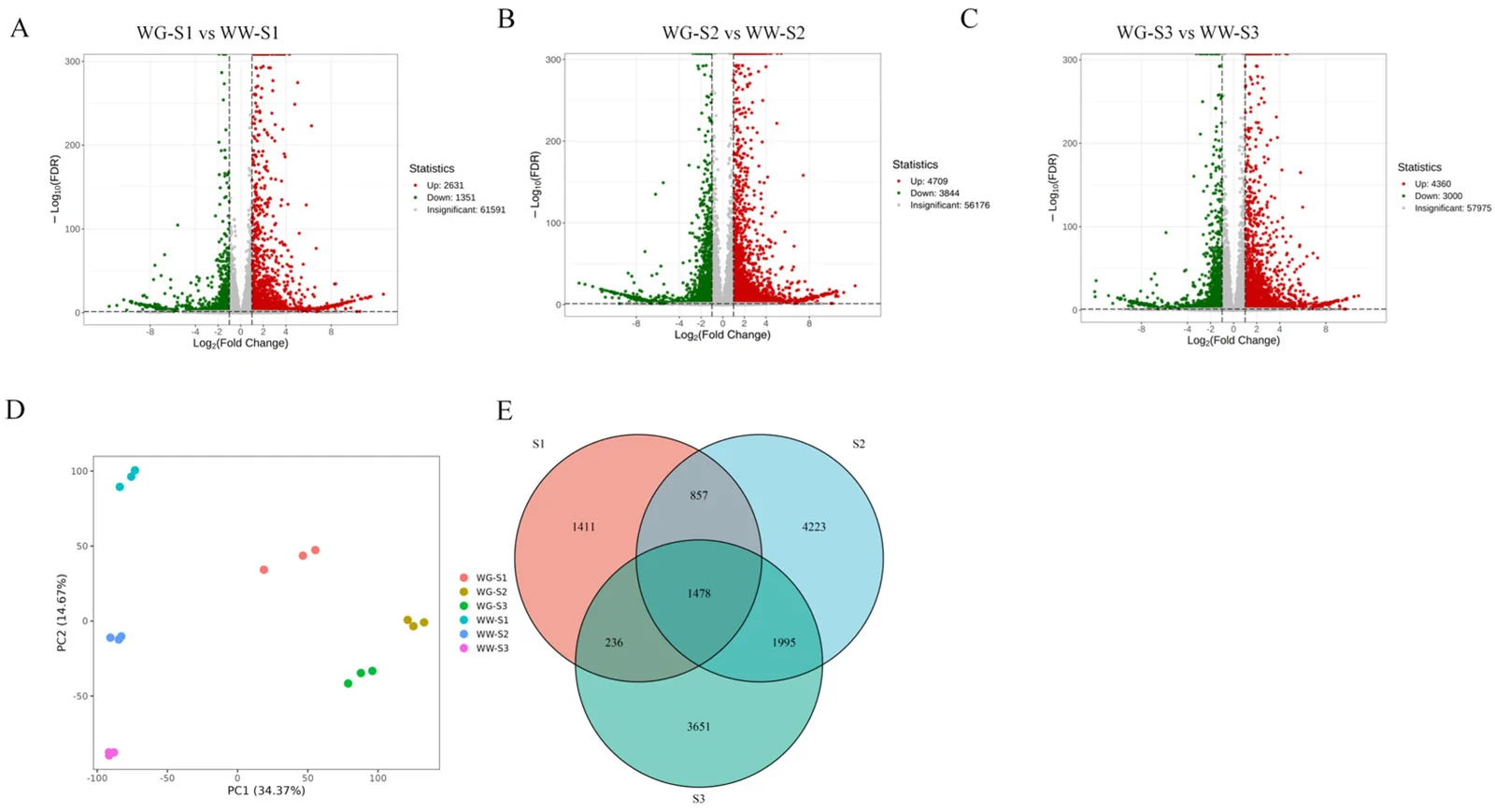
Transcriptome analysis reveals transcriptional differences between fully green (WG) and fully white (WW) leaf bud phenotypes during leaf development of *Sinobambusa tootsik* f. *albostriata*. (**A**) Volcano plot of DEGs between WG and WW at S1 stage. (**B**) Volcano plot of DEGs between WG and WW at S2 stage. (**C**) Volcano plot of DEGs between WG and WW at S3 stage. (**D**) PCA analysis of transcriptome samples. (**E**) Venn diagram showing stage-specific and shared DEGs among different developmental stages. Dashed lines indicate thresholds for identifying differentially expressed genes (DEGs), with vertical dashed lines representing |log_2_(fold change)| = 1 and horizontal dashed line representing FDR = 0.05.

Principal component analysis (PCA) was performed using 18 transcriptome samples (Figure 1D). The first and second principal components explained 34.37% and 14.67% of the total variance, respectively. All samples were clearly separated into six groups according to phenotype and developmental stage. Biological replicates within the same phenotype and developmental stage clustered closely, indicating high reproducibility. In contrast, samples from different phenotypes or developmental stages exhibited distinct separation patterns. Within the same phenotype, samples from adjacent developmental stages showed relatively closer clustering distances, whereas WG and WW samples at the same developmental stage displayed distinct spatial distributions. These results indicated substantial differences in transcriptional profiles between the two leaf color phenotypes during development. Pearson correlation analysis further revealed strong correlations among biological replicates, confirming the reliability and reproducibility of the transcriptome dataset (Figure S1).

To identify genes associated with leaf color differentiation, differentially expressed gene (DEG) analysis was conducted between WG and WW leaf buds at the same developmental stage, including WG-S1 vs. WW-S1, WG-S2 vs. WW-S2, and WG-S3 vs. WW-S3. The WG-S2 vs. WW-S2 comparison exhibited the largest number of DEGs, with 4709 up-regulated and 3844 down-regulated genes (Figure 1B). In comparison, fewer DEGs were detected in the WG-S1 vs. WW-S1 and WG-S3 vs. WW-S3 groups, which contained 2631 up-regulated/1351 down-regulated genes and 4360 up-regulated/3000 down-regulated genes, respectively (Figure 1A,C). These results indicated that transcriptional differences between WG and WW phenotypes varied across developmental stages, with the greatest divergence occurring at the S2 stage.

Stage-specific expression patterns of DEGs were further analyzed among the three developmental stages using a TPM threshold >1 (Figure 1E). A total of 1411 genes exhibited stage-specific expression at the S1 stage, whereas 4223 and 3651 genes were specifically expressed at the S2 and S3 stages, respectively. In addition, 857 genes were uniquely shared between the S1 and S2 stages, 236 genes were shared between the S1 and S3 stages, and 1995 genes were specifically coexpressed between the S2 and S3 stages. Furthermore, 1478 genes showed stable expression across all three developmental stages. These results demonstrated that gene expression patterns in *S. tootsik* f. *albostriata* leaf buds displayed strong developmental stage specificity, suggesting that distinct developmental stages involve the activation of stage-specific regulatory networks associated with leaf development and color formation.

### 2.2. Functional Enrichment Analysis

**Figure 2:**
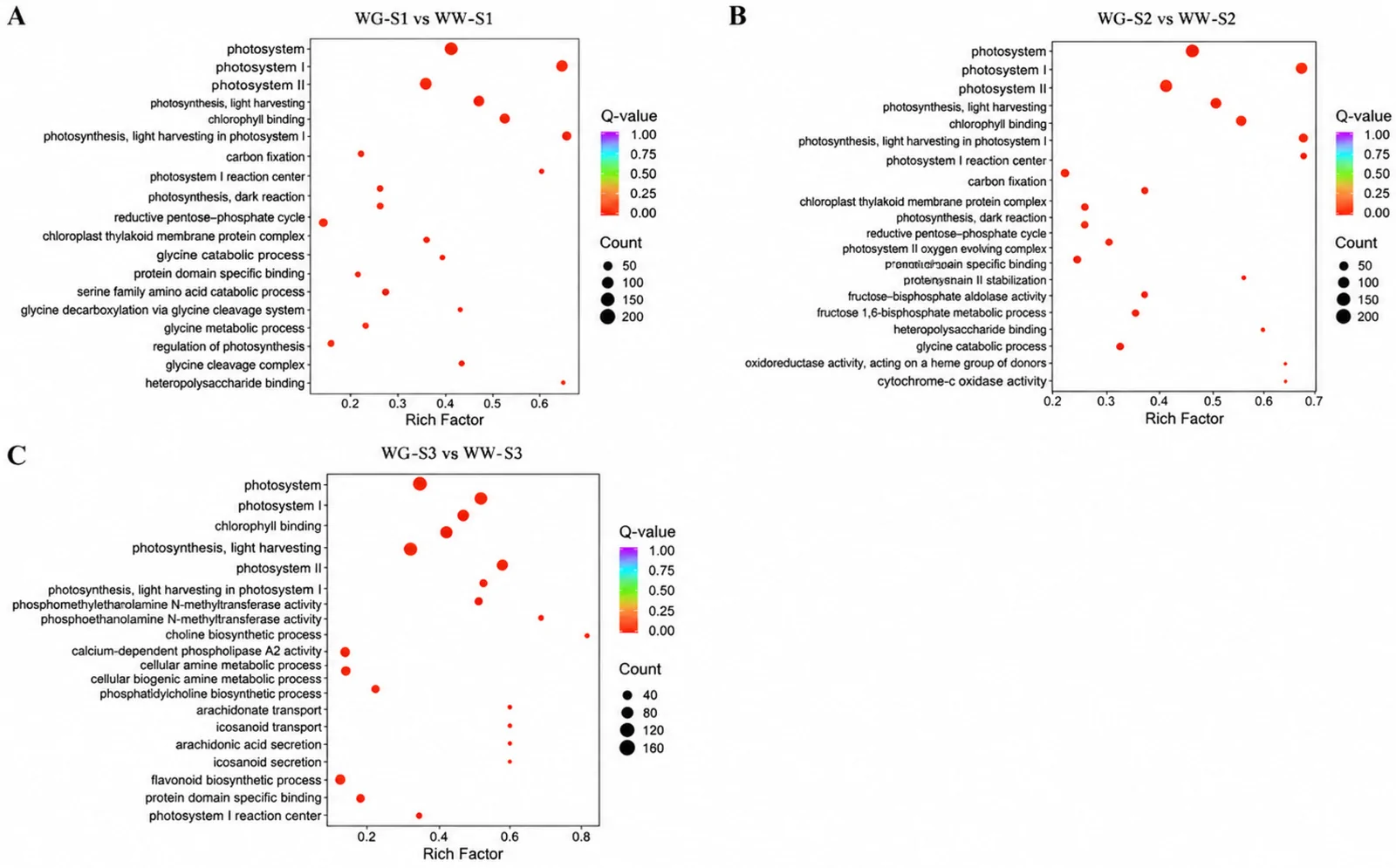
GO enrichment analysis of differentially expressed genes (DEGs) among three comparison groups. (**A**) GO enrichment analysis of DEGs in the WG-S1 vs. WW-S1 comparison. (**B**) GO enrichment analysis of DEGs in the WG-S2 vs. WW-S2 comparison. (**C**) GO enrichment analysis of DEGs in the WG-S3 vs. WW-S3 comparison. The x-axis represents the Rich Factor, with higher values indicating stronger enrichment of the corresponding GO terms. Dot colors represent the Q-values (adjusted *p*-values), with red indicating lower Q-values and higher statistical significance. Dot sizes represent the number of DEGs (count) associated with each GO term, with larger dots indicating a greater number of enriched genes.

Gene Ontology (GO) enrichment analysis was performed on the DEGs identified from the three comparison groups (Figure 2). Several photosynthesis-related GO terms were significantly enriched across all three comparisons, including photosynthesis, light harvesting, photosystem I, and chlorophyll binding. These enriched terms were closely associated with chloroplast function, photosynthetic apparatus assembly, and pigment accumulation.

In the WG-S1 vs. WW-S1 and WG-S2 vs. WW-S2 comparisons, DEGs were predominantly enriched in pathways related to photosynthetic machinery, including photosystem I, photosystem II, chlorophyll binding, photosynthetic light harvesting, carbon fixation, and chloroplast thylakoid membrane protein complexes (Figure 2A,B). In the WG-S3 vs. WW-S3 comparison, photosynthesis-related terms remained significantly enriched (Figure 2C). However, additional GO terms associated with lipid metabolism, hormone-related molecular functions, and secondary metabolic processes were also identified, including calcium-dependent phospholipase A2 activity, isoprenoid transport, and flavonoid biosynthetic processes.

Collectively, these results indicated that alterations in photosynthetic apparatus organization, chlorophyll binding, and light-harvesting processes represent major transcriptional differences between WG and WW leaf buds throughout development. During the early developmental stages (S1 and S2), transcriptional divergence between the two phenotypes was mainly associated with photosynthetic system establishment and chloroplast development. At the later S3 stage, additional differences involving secondary metabolism, lipid metabolism, and hormone-related pathways emerged, suggesting that multiple metabolic processes may contribute to the maintenance of leaf color phenotypes in *S. tootsik* f. *albostriata*.

### 2.3. Analysis of Chlorophyll Content

**Figure 3:**
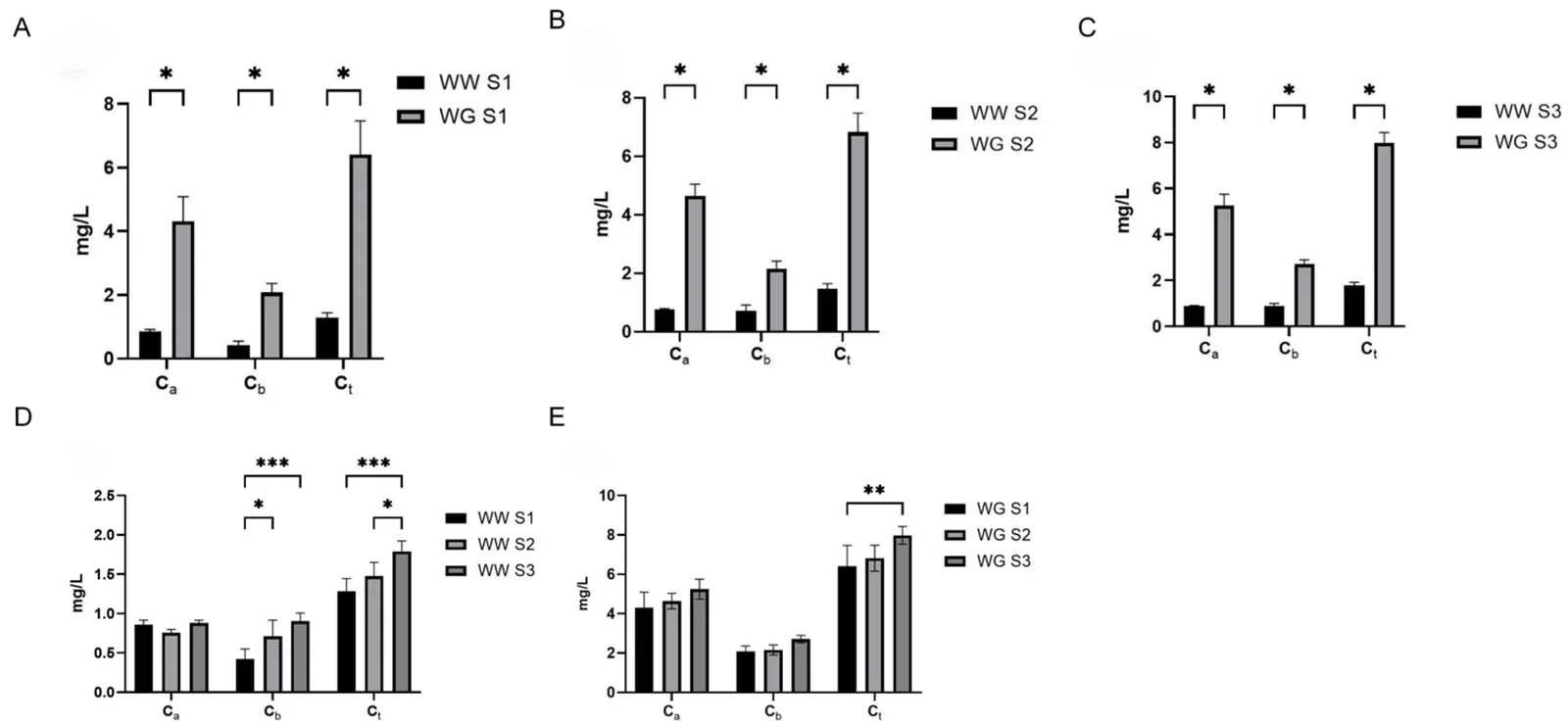
Determination of chlorophyll contents in WG and WW leaf buds. (**A**) Chlorophyll contents in WG and WW leaf buds at the S1 developmental stage. (**B**) Chlorophyll contents in WG and WW leaf buds at the S2 developmental stage. (**C**) Chlorophyll contents in WG and WW leaf buds at the S3 developmental stage. (**D**) Changes in chlorophyll contents of WG leaf buds across the three developmental stages (S1–S3). (**E**) Changes in chlorophyll contents of WW leaf buds across the three developmental stages (S1–S3). C_a_, chlorophyll a content; C_b_, chlorophyll b content; C_t_, total chlorophyll content (mg/g). Asterisks indicate statistical significance (* *p* < 0.05, ** *p* < 0.01, and *** *p* < 0.001).

Chlorophyll contents were determined in WG and WW leaf buds at three developmental stages (S1, S2, and S3) (Figure 3). The results showed that the contents of chlorophyll a, chlorophyll b, and total chlorophyll were significantly higher in WG than in WW leaf buds at all examined developmental stages.

At the S1 stage, the total chlorophyll content reached 0.32 mg/g in WG leaf buds, whereas it was only 0.06 mg/g in WW leaf buds. At the S2 stage, total chlorophyll content increased slightly to 0.34 mg/g in WG, while WW maintained a low level of 0.07 mg/g. By the S3 stage, total chlorophyll content further increased to 0.40 mg/g in WG, whereas WW remained at only 0.09 mg/g. Significant differences in both chlorophyll a and chlorophyll b contents were observed between WG and WW at each developmental stage. These results demonstrated that WW leaf buds exhibited a substantially reduced capacity for chlorophyll accumulation compared with WG leaf buds, resulting in the distinct green and white phenotypes.

### 2.4. Analysis of CPOX Enzyme Activity

**Figure 4:**
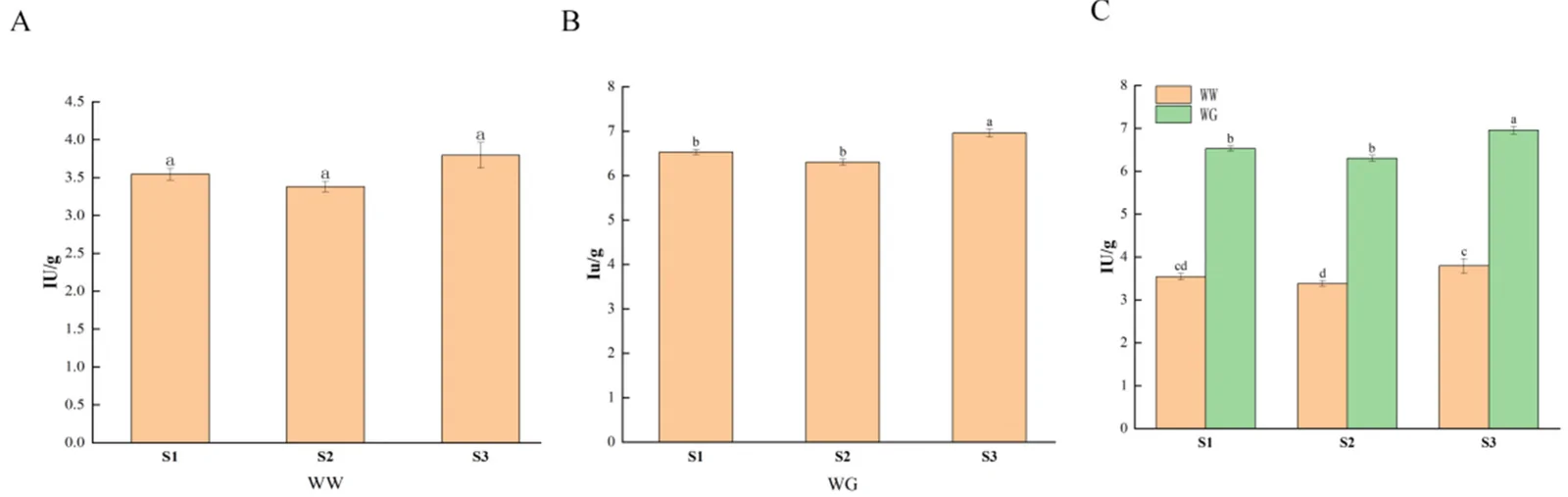
Determination of CPOX enzyme activity in WG and WW leaf buds. (**A**) CPOX enzyme activity in WG leaf buds at three developmental stages (S1–S3). (**B**) CPOX enzyme activity in WW leaf buds at three developmental stages (S1–S3). (**C**) Comparison of CPOX enzyme activity between WG and WW leaf buds at the same developmental stage. Different lowercase letters indicate significant differences (*p* < 0.05).

CPOX enzyme activity was further measured to evaluate differences in chlorophyll biosynthetic capacity between the two phenotypes (Figure 4). Although the developmental trends of CPOX activity were similar in WG and WW leaf buds, the activity levels in WW were consistently lower than those in WG throughout development. In WW leaf buds, CPOX activity was 3.544 IU/g at the S1 stage, decreased slightly to 3.380 IU/g at the S2 stage, and increased significantly to 3.794 IU/g at the S3 stage. The CPOX activity at S3 was significantly higher than that at S1 and S2, indicating that CPOX activity gradually increased during WW leaf bud development, despite remaining at a relatively low level.

In WG leaf buds, CPOX activity showed an overall increasing trend during development. The activity was 6.530 IU/g at S1, decreased slightly to 6.303 IU/g at S2, and reached the highest level of 6.958 IU/g at S3. No significant difference was detected between S1 and S2, whereas CPOX activity at S3 was significantly higher than that at the earlier developmental stages. These results indicated that the enhancement of CPOX catalytic activity during later developmental stages may contribute to increased chlorophyll accumulation in WG leaf buds.

### 2.5. Screening of StaHemF Candidate Genes

Based on transcriptome data and multiple rounds of screening, six candidate HemF genes were identified from *S. tootsik* f. *albostriata*. These candidate genes were designated as *StaHemF1–StaHemF6* and were subjected to further bioinformatic and expression analyses (Table 1)

**Figure 5:**
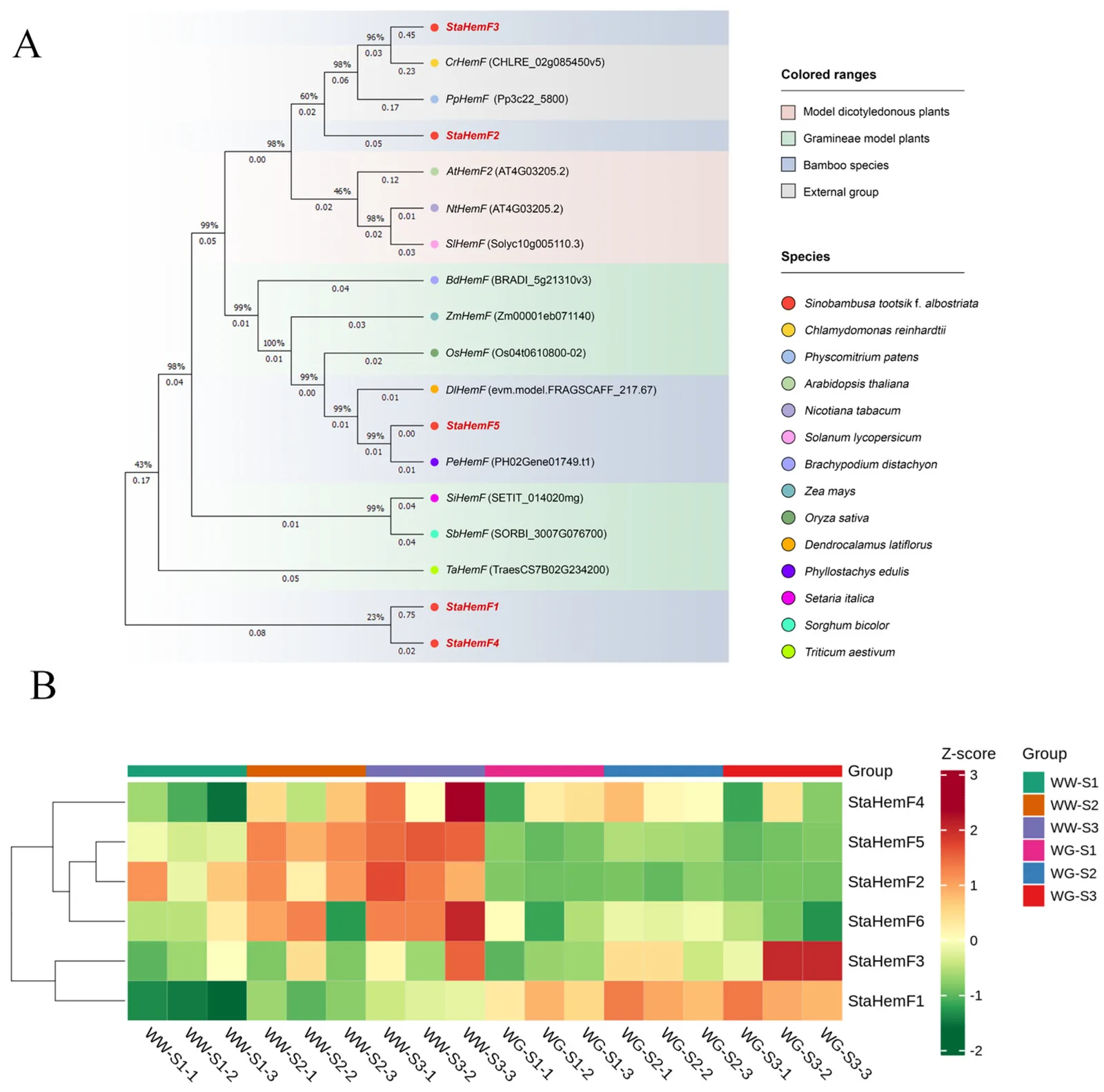
Phylogenetic analysis and expression patterns of *StaHemF* genes in Sinobambusa tootsik f. albostriata. (**A**) Phylogenetic tree of candidate *StaHemF* proteins from *S. tootsik* f. *albostriata* and homologous HemF proteins from representative plant species. The analyzed species included model dicotyledonous plants, Gramineae model plants, bamboo species, and an external group. (**B**) Expression heatmap showing the transcriptional patterns of *StaHemF* genes during different developmental stages of *S. tootsik* f. *albostriata*. The species used for phylogenetic analysis included *Sinobambusa tootsik* f. *albostriata*, *Physcomitrium patens*, *Chlamydomonas reinhardtii*, *Arabidopsis thaliana*, *Nicotiana tabacum*, *Solanum lycopersicum*, *Brachypodium distachyon*, *Zea mays*, *Oryza sativa, Dendrocalamus latiflorus*, *Phyllostachys edulis*, *Setaria italica, Sorghum bicolor*, and *Triticum aestivum.*

To investigate the evolutionary relationships and potential functional divergence of candidate *StaHemF* genes in *S. tootsik* f. *albostriata*, a phylogenetic analysis was performed using HemF protein sequences from model dicotyledonous plants, Gramineae model plants, bamboo species, and an external group (Figure 5A). The phylogenetic tree revealed that the HemF proteins from green algae and mosses were located at the basal position, indicating their early evolutionary divergence. The HemF proteins from higher plants were mainly clustered into three distinct groups corresponding to dicots, grasses, and bamboos, suggesting lineage-specific differentiation during plant evolution.

HemF proteins from grass species, which have been functionally characterized as important components of chlorophyll biosynthesis and chloroplast development, formed a closely related clade. Notably, *StaHemF5* clustered with functionally characterized HemF proteins from rice, maize, and *Dendrocalamus latiflorus*, with a bootstrap value close to 100% at the corresponding node. This close evolutionary relationship suggested that *StaHemF5* retains highly conserved sequence features and may possess a similar CPOX-related function in chlorophyll biosynthesis. In contrast, other *StaHemF* members exhibited relatively distant evolutionary relationships with the characterized grass HemF proteins, and several internal branches showed lower bootstrap support values, suggesting possible functional diversification among these members. *StaHemF6* was excluded from phylogenetic analysis due to unreliable sequence alignment and showed the lowest degree of evolutionary conservation among the identified candidates.

The expression patterns of *StaHemF* genes were further analyzed using transcriptome data (Figure 5B). Among the six candidate genes, *StaHemF5* exhibited distinct differential expression patterns between WW and WG leaf buds. Specifically, *StaHemF5* showed relatively high expression levels in WW leaf buds throughout the three developmental stages (S1–S3), whereas its expression remained relatively low in WG leaf buds. Considering that WW leaf buds exhibited significantly reduced CPOX activity and chlorophyll contents compared with WG leaf buds, the differential expression pattern of *StaHemF5* suggested a close association between this gene and the chlorophyll-deficient phenotype in *S. tootsik* f. *albostriata*. The other *StaHemF* members displayed weaker or less consistent expression differences between WW and WG leaf buds. For example, *StaHemF1* showed relatively high expression in WW at early developmental stages but declined subsequently. *StaHemF2*, *StaHemF3*, and *StaHemF4* did not exhibit obvious phenotype-specific or developmental-stage-dependent expression patterns, making their potential contributions to leaf color variation less evident.

Taken together, the combination of phylogenetic conservation and differential expression analysis identified *StaHemF5* as the most promising candidate associated with chlorophyll accumulation and leaf color differentiation. The conserved evolutionary position of *StaHemF5* within the HemF family, together with its distinct expression pattern between WW and WG leaf buds, supports its potential involvement in regulating chlorophyll biosynthesis. Therefore, *StaHemF5* was selected for further bioinformatic characterization and functional validation through transient overexpression in tobacco.

### 2.6. Bioinformatic Characterization of the StaHemF5 Protein

#### 2.6.1. Physicochemical Property Analysis of StaHemF Amino Acid Sequence

The physicochemical properties of the StaHemF protein were analyzed to characterize its basic molecular features (Table 2). The results showed that StaHemF has a predicted molecular weight of 43,501.02 Da and a theoretical molecular formula of C_1953_H_2976_N_544_O_569_S_10_. The theoretical isoelectric point (pI) was calculated as 6.48. The protein sequence contains both basic and acidic amino acid residues, including 49 basic residues (Arg and Lys) and 51 acidic residues (Asp and Glu), indicating that StaHemF is a weakly acidic protein (Table 3). According to the instability index, StaHemF was predicted to be an unstable protein. Amino acid composition analysis revealed that StaHemF contains all 20 standard amino acids, with alanine (Ala) and glycine (Gly) being the most abundant residues, accounting for 9.5% and 9.2% of the total amino acid composition, respectively.

#### 2.6.2. Protein Sequence Analysis of StaHemF

**Figure 6:**
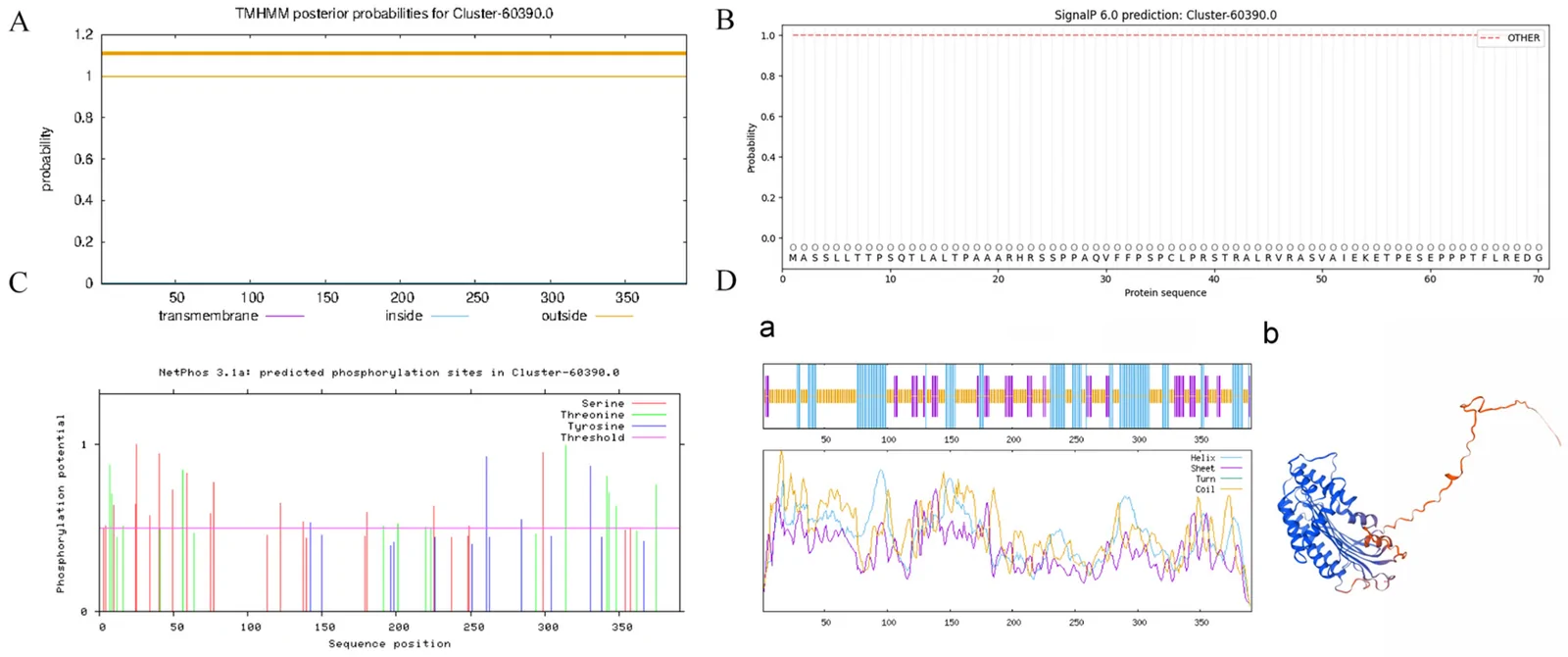
Bioinformatic analysis of the sequence and structural characteristics of the StaHemF protein. (**A**) Prediction of transmembrane regions in the StaHemF protein sequence. The yellow line indicates the predicted signal peptide region. (**B**) Prediction of the signal peptide sequence of the StaHemF protein. (**C**) Prediction of potential phosphorylation sites in the StaHemF protein. (**D**) Structural analysis of StaHemF protein, including (**a**) secondary structure prediction and (**b**) tertiary structure modeling. Blue represents α-helices; green represents β-turns; purple represents β-sheets; yellow represents coils.

The transmembrane structure and signal peptide characteristics of StaHemF were further analyzed. Transmembrane domain prediction indicated that StaHemF lacks typical transmembrane regions, suggesting that it is a soluble non-transmembrane protein (Figure 6A). Signal peptide analysis further showed that StaHemF does not contain a classical N-terminal secretory signal peptide sequence (Figure 6B). Prediction of phosphorylation sites revealed that StaHemF contains 53 potential phosphorylation sites, including 29 serine residues, 18 threonine residues, and 6 tyrosine residues (Figure 6C). These results suggest that StaHemF may undergo multiple phosphorylation modifications, which could contribute to the regulation of its protein activity. The secondary and tertiary structures of StaHemF were predicted to further investigate its structural characteristics (Figure 6D). The StaHemF protein consists of 391 amino acid residues and exhibits typical multi-element secondary structural features. The predicted secondary structure includes 127 residues forming α-helices, 23 residues forming β-turns, 64 residues forming extended strands, and 177 residues forming random coils. Among these structural components, α-helices and random coils represent the predominant structural elements of StaHemF.

#### 2.6.3. Subcellular Localization Prediction of StaHemF

Subcellular localization prediction based on the amino acid sequence indicated that StaHemF is mainly targeted to chloroplasts, consistent with its potential involvement in chlorophyll biosynthesis (Table 4).

#### 2.6.4. Sequence Homology Analysis of StaHemF

**Figure 7:**
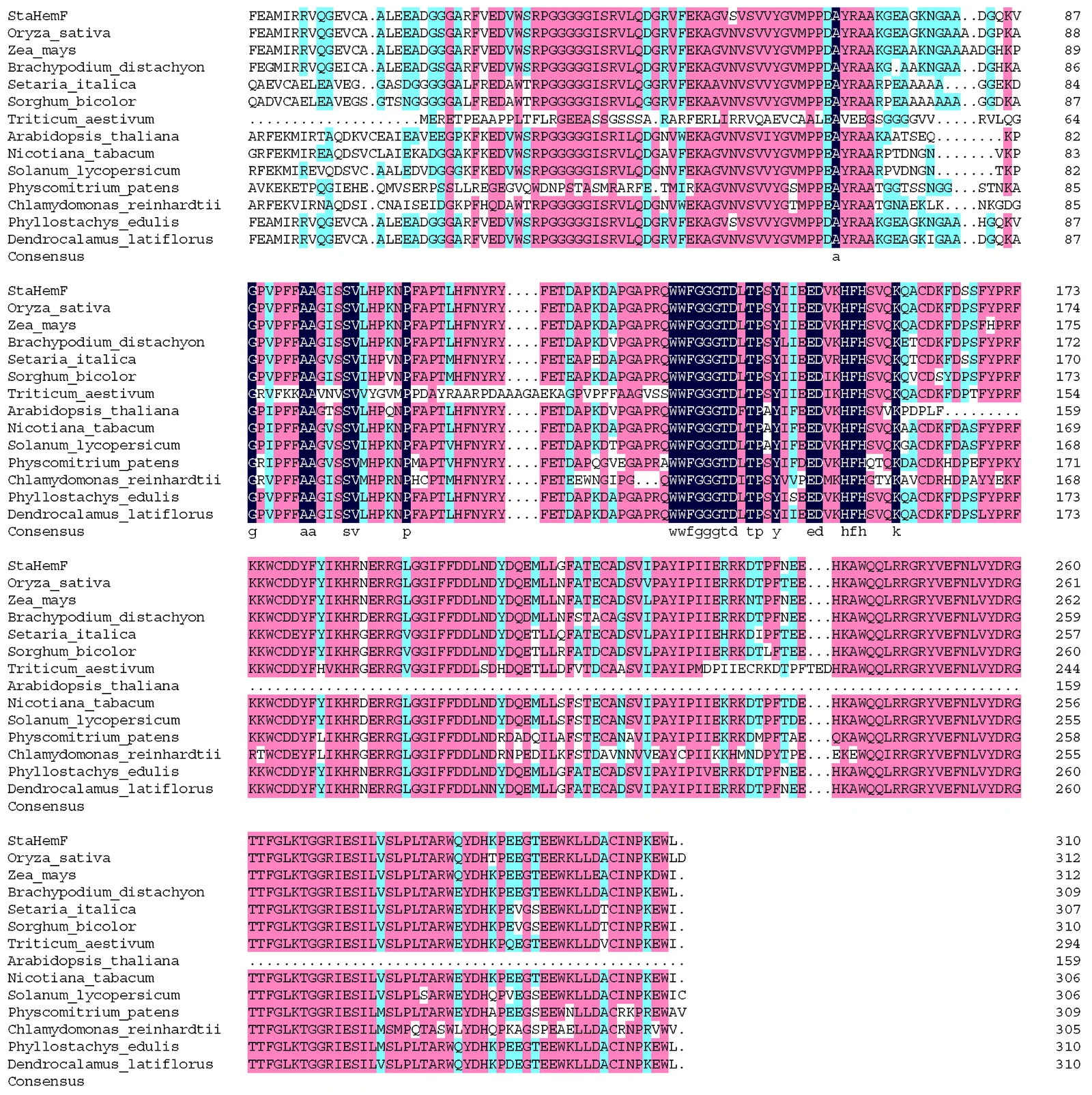
Amino acid sequence alignment of StaHemF with other plants.

Multiple sequence alignment analysis revealed that StaHemF shares extensive amino acid conservation with homologous HEMF proteins from different plant species (Figure 7). The homologous HEMF proteins exhibited high sequence similarity, with an overall identity of 77.60%. Compared with representative model plants, including *Arabidopsis thaliana*, *Oryza sativa*, and *Zea mays*, StaHemF showed sequence similarity values exceeding 85%. The highest sequence identities were observed with *Phyllostachys edulis* and *Dendrocalamus latiflorus*, reaching 94.72% and 94.10%, respectively. Furthermore, the core functional regions of HEMF proteins contained multiple highly conserved amino acid motifs, and StaHemF displayed minimal sequence variation compared with HEMF homologs from other plant species. No large-scale deletions or substitutions were observed within these conserved regions. These conserved domains are generally associated with enzyme catalytic activity and structural stability, suggesting that StaHemF may retain biological functions similar to HEMF proteins from other plant species.

### 2.7. Analysis of StaHemF Gene Expression Specificity

**Figure 8:**
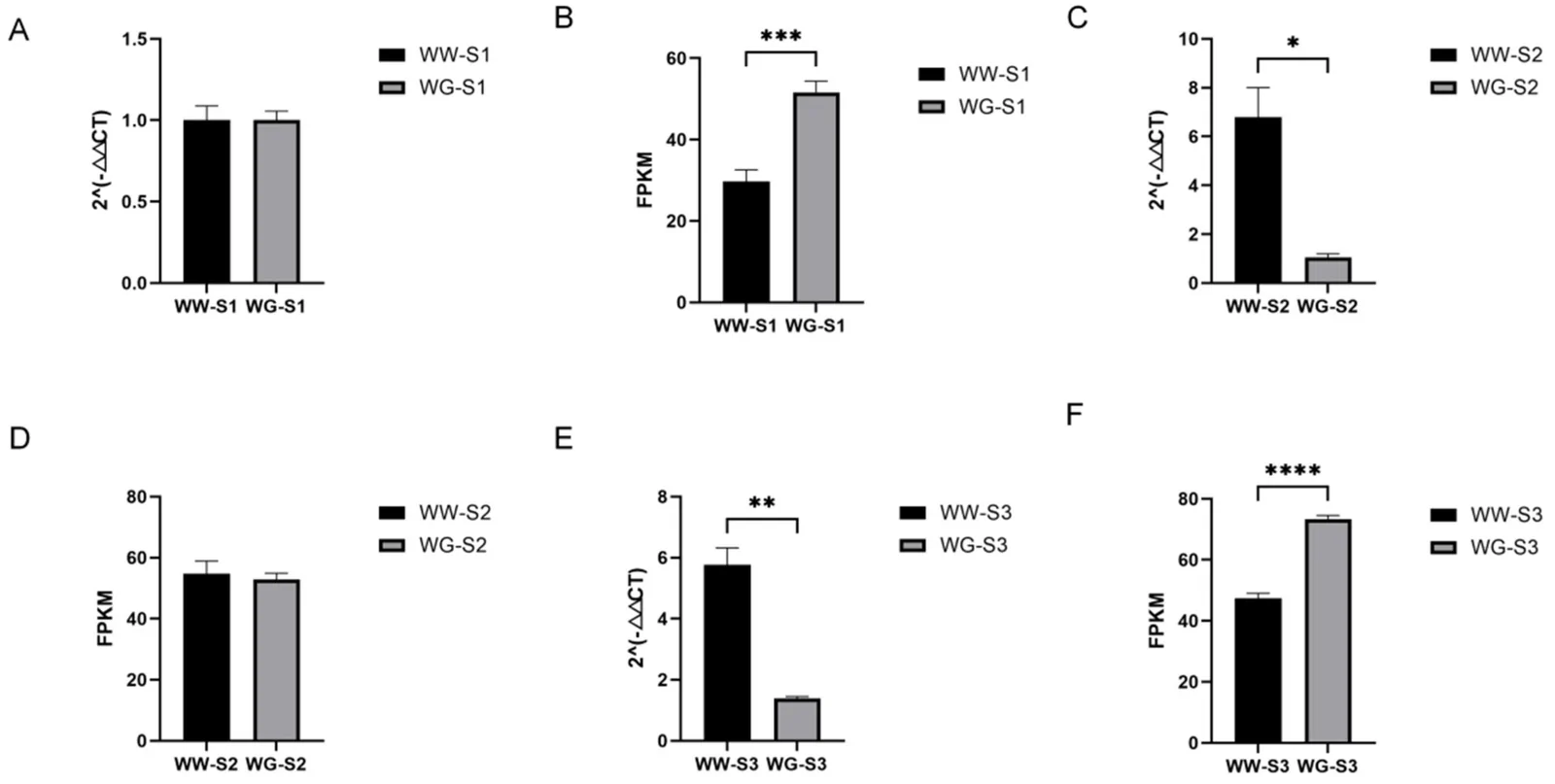
Expression patterns of *StaHemF* in WW and WG leaf buds at three developmental stages. (**A**,**C**,**E**) Relative expression levels of *StaHemF* in WW and WG leaf buds at the S1, S2, and S3 developmental stages determined by qRT-PCR using the 2^−ΔΔCt^ method. (**B**,**D**,**F**) Transcript abundance of *StaHemF* based on FPKM values obtained from RNA-seq data. S1–S3 represent successive developmental stages of leaf buds. Asterisks indicate statistical significance (* *p* < 0.05, ** *p* < 0.01, *** *p* < 0.001, and **** *p* < 0.0001).

The expression pattern of *StaHemF* was analyzed by qRT-PCR and compared with transcriptome data at three developmental stages (S1–S3) (Figure 8). At the S1 stage, qRT-PCR analysis showed no significant difference in *StaHemF* expression between WW and WG leaf buds (Figure 8A). However, transcriptome data indicated that the transcript abundance of *StaHemF* was significantly higher in WG-S1 than in WW-S1 (Figure 8B). At the S2 stage, qRT-PCR results showed that *StaHemF* expression was significantly higher in WW than in WG (Figure 8C), whereas the FPKM values from transcriptome analysis showed no significant difference between the two phenotypes (Figure 8D). Nevertheless, both datasets displayed a similar developmental expression trend. At the S3 stage, qRT-PCR results continued to show significantly higher *StaHemF* expression in WW leaf buds than in WG leaf buds (Figure 8E). In contrast, transcriptome analysis showed that *StaHemF* transcript abundance was significantly higher in WG than in WW (Figure 8F), revealing an opposite expression pattern between the two approaches.

Overall, qRT-PCR analysis showed that *StaHemF* expression gradually increased in WW relative to WG during leaf bud development, whereas transcriptome data consistently showed higher transcript abundance in WG from S1 to S3 stages. The discrepancy between qRT-PCR and RNA-seq results became more pronounced at later developmental stages.

**Figure 9:**
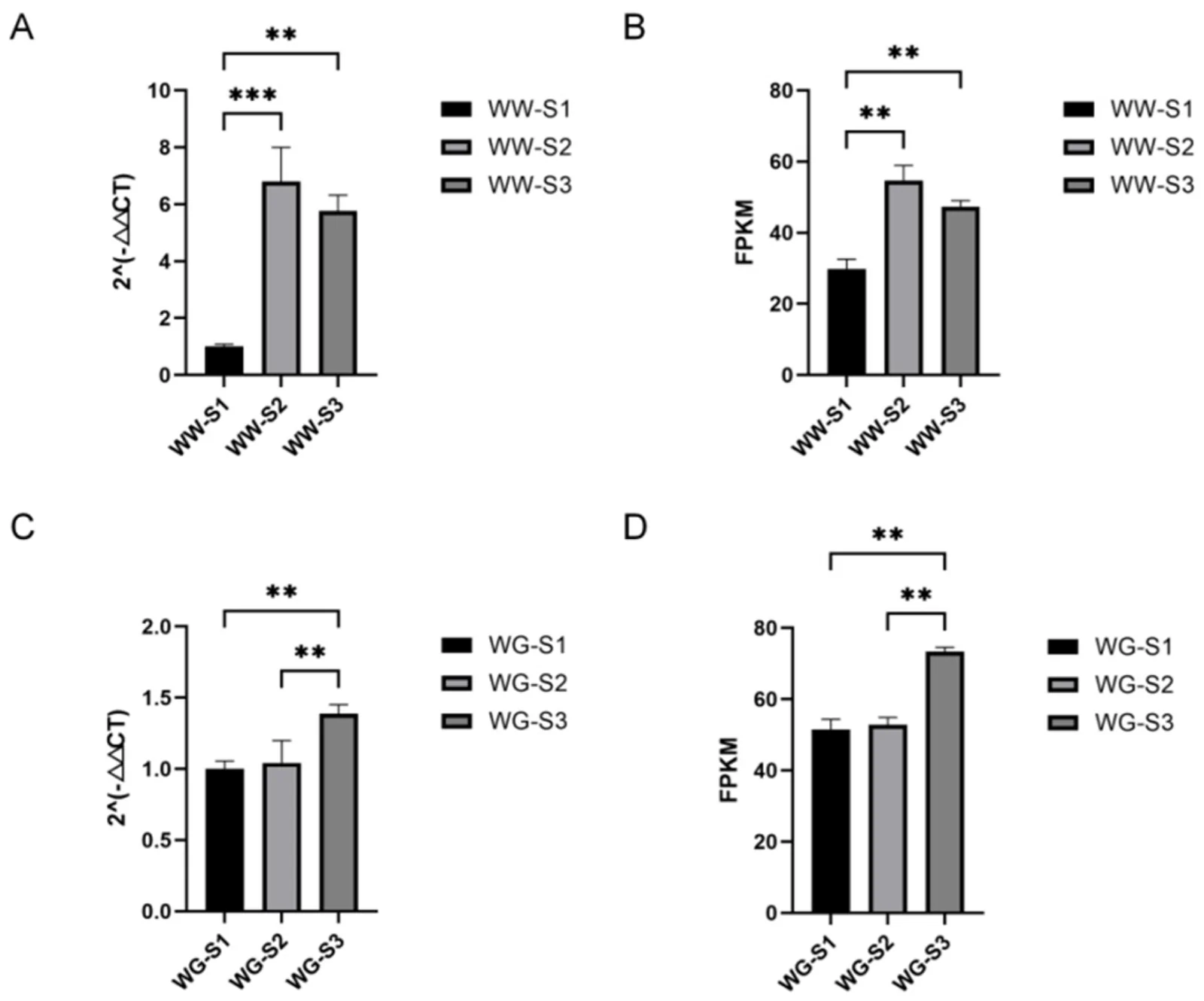
Temporal expression patterns of *StaHemF* within WW and WG leaf buds during development. (**A**) Relative expression levels of *StaHemF* in WW leaf buds at the S1, S2, and S3 developmental stages determined by qRT-PCR using the 2^−ΔΔCt^ method. (**B**) Transcript abundance of *StaHemF* in WW leaf buds at three developmental stages based on RNA-seq FPKM values. (**C**) Relative expression levels of *StaHemF* in WG leaf buds at the S1, S2, and S3 developmental stages determined by qRT-PCR. (**D**) Transcript abundance of *StaHemF* in WG leaf buds during development based on RNA-seq FPKM values. S1–S3 represent successive leaf bud developmental stages. Asterisks indicate statistical significance (** *p* < 0.01, *** *p* < 0.001).

The developmental expression patterns of *StaHemF5* within each phenotype were further analyzed. In WW leaf buds, both qRT-PCR and transcriptome data showed that *StaHemF* expression increased significantly from S1 to S2, reached the highest level at S2, and then slightly decreased at S3. The expression levels at S2 and S3 were significantly higher than those at S1, indicating an overall increase followed by a moderate decline during WW development (Figure 9A,B). In WG leaf buds, qRT-PCR results showed that *StaHemF* expression was significantly up-regulated only at the S3 stage, while expression levels at S1 and S2 remained relatively stable. Consistently, transcriptome data showed similar transcript abundance between WG-S1 and WG-S2, followed by a substantial increase at WG-S3 (Figure 9C,D).

Taken together, *StaHemF* exhibited distinct developmental expression patterns between WW and WG leaf buds. The induction of *StaHemF* expression from S1 to S2 occurred earlier and more prominently in WW, whereas its up-regulation in WG mainly occurred during the later S3 developmental stage. These results suggested that *StaHemF* may respond differently to developmental signals between the two leaf color phenotypes.

### 2.8. Subcellular Localization

**Figure 10:**
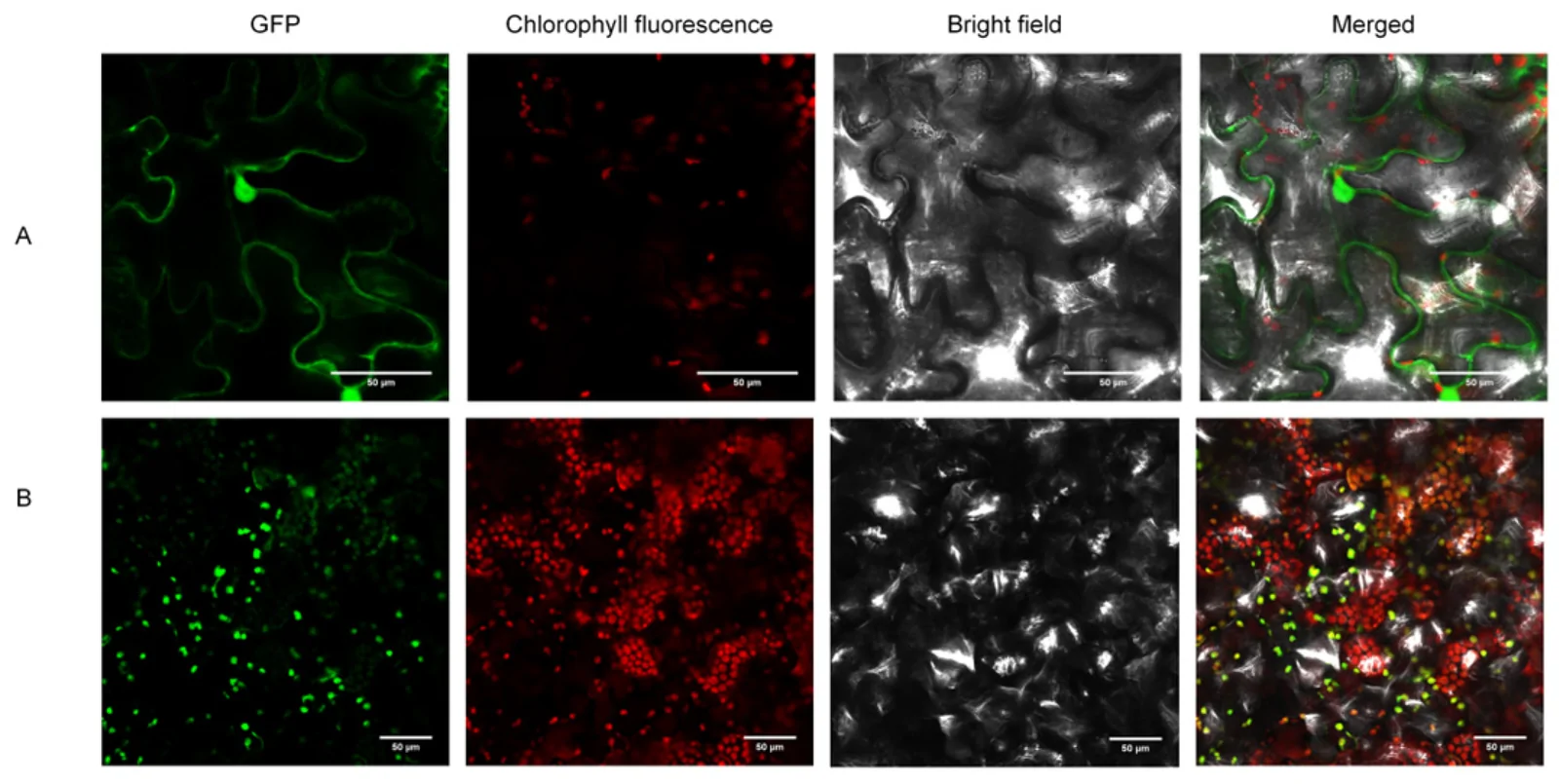
Subcellular localization of StaHemF protein in tobacco leaf cells. (**A**) Subcellular localization of the empty vector pCAMBIA1302 expressed in *Nicotiana benthamiana* leaf tissues. (**B**) Subcellular localization of the recombinant construct pCAMBIA1302-*StaHemF*-GFP transiently expressed in *N. benthamiana* leaf tissues. Green fluorescence indicates GFP signals, while red fluorescence represents chlorophyll autofluorescence from chloroplasts. The merged images show the overlap between GFP and chlorophyll fluorescence signals. Scale bars = 50 μm.

To determine the subcellular localization of *StaHemF* protein, the recombinant vector pCAMBIA1302-*StaHemF*-GFP and the empty vector pCAMBIA1302 were transiently introduced into tobacco leaf cells. The GFP fluorescence signal of *StaHemF*-GFP was mainly observed in chloroplasts, consistent with the subcellular localization prediction. These results indicated that *StaHemF* is localized in chloroplasts and may function in chloroplast-associated biological processes (Figure 10).

### 2.9. Functional Validation of StaHemF Overexpression in Tobacco

**Figure 11:**
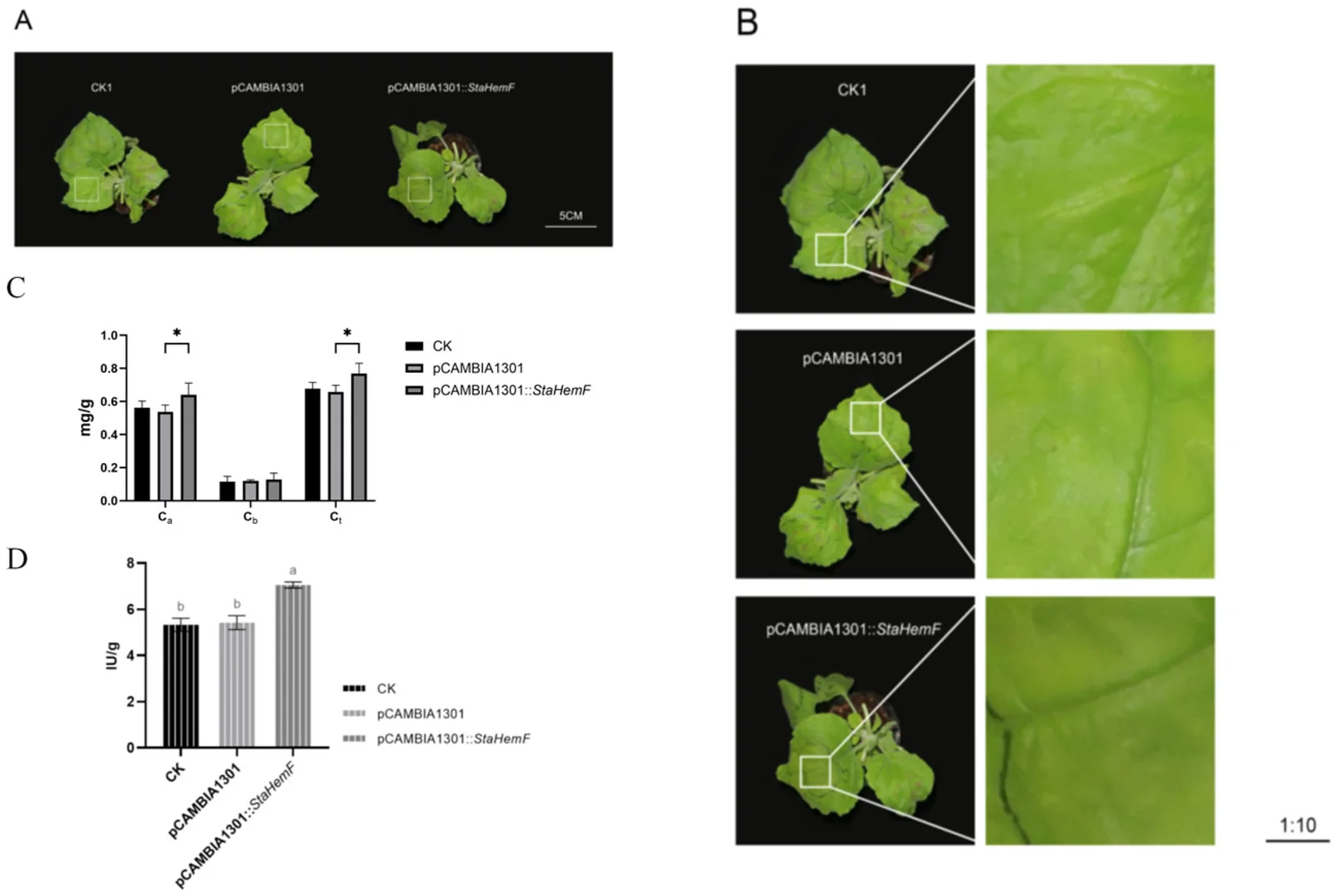
Functional analysis of *StaHemF* through transient overexpression in *Nicotiana benthamiana*. (**A**,**B**) Phenotypic effects of transient *StaHemF* overexpression in *N. benthamiana* leaves. (**C**) Contents of chlorophyll a, chlorophyll b, and total chlorophyll in tobacco leaves after transient infiltration. (**D**) CPOX enzyme activity in tobacco leaves after transient infiltration. C_a_, chlorophyll a content; C_b_, chlorophyll b content; C_t_, total chlorophyll content. Asterisks and different lowercase letters indicate statistically significant differences between compared groups (*p* < 0.05).

To further investigate the biological function of *StaHemF*, the recombinant overexpression vector pCAMBIA1301-*StaHemF* and the empty vector pCAMBIA1301 were transiently expressed in tobacco leaves. Compared with the control treatments, including non-infiltrated leaves (CK) and empty vector controls, tobacco leaves overexpressing *StaHemF* exhibited a slightly enhanced green coloration. No obvious phenotypic differences were observed between the empty vector treatment and CK groups (Figure 11A,B).

Further physiological analysis revealed that transient overexpression of *StaHemF* significantly increased chlorophyll accumulation in tobacco leaves. Compared with CK and empty vector controls, *StaHemF*-overexpressing leaves exhibited significantly higher levels of chlorophyll a, chlorophyll b, and total chlorophyll contents. Among all treatments, the overexpression group showed the highest chlorophyll a and total chlorophyll levels, with significant differences detected at *p* < 0.05. No significant differences were observed between the empty vector and CK groups, indicating that vector transformation itself did not affect chlorophyll accumulation (Figure 11C).

CPOX activity was further measured in tobacco leaves following transient expression (Figure 11D). *StaHemF* overexpression significantly enhanced CPOX enzyme activity compared with CK and empty vector controls, whereas no significant difference was observed between the empty vector and CK groups. These results demonstrated that heterologous expression of *StaHemF* effectively increased CPOX activity and suggested that *StaHemF* positively contributes to chlorophyll biosynthesis through regulation of the porphyrin metabolic pathway (Figure 11D)

## 3. Discussion

### 3.1. Abnormal Photosynthetic Activity and Chlorophyll Metabolism Underlie the Albino Phenotype of S. tootsik f. albostriata

Transcriptome analysis revealed that DEGs between WG and WW leaf buds at different developmental stages were predominantly enriched in photosynthesis-related pathways, including photosystem I, photosystem II, chlorophyll-binding proteins, and carbon fixation. This expression pattern is consistent with previous reports on other chlorophyll-deficient bamboo species, suggesting that disruption of the photosynthetic apparatus represents a conserved molecular basis underlying leaf color variation in bamboo [2,6,15,16,17]. Proteomic and physiological studies in diverse plant species have further demonstrated that coordinated down-regulation of photosynthetic proteins and abnormal chloroplast ultrastructure are common features associated with albino and chlorotic leaf phenotypes [18,19,20]. Combined with our physiological results showing significantly reduced chlorophyll contents in WW tissues, these findings indicate that impaired photosynthetic capacity and defective chloroplast development are key factors contributing to the albino phenotype of *S. tootsik* f. *albostriata*. Similar alterations in photosynthetic machinery and chloroplast function have also been widely observed during albino leaf formation in other plant species, including bamboo.

Notably, the S2 developmental stage exhibited the highest number of DEGs and contained a large proportion of stage-specific expressed genes, indicating that this period of rapid leaf bud expansion may represent a critical developmental window for leaf color differentiation. As leaf development progressed, the enrichment of photosynthesis-related pathways gradually decreased, whereas genes associated with secondary metabolism and signal transduction became increasingly prominent. This transition suggests that early developmental stages are mainly characterized by disturbances in photosynthetic establishment, while later stages involve more complex regulatory networks that maintain the divergence between green and white phenotypes. Nevertheless, the persistent disruption of chlorophyll metabolism throughout all examined stages highlights its fundamental importance in determining the albino phenotype of *S. tootsik* f. *albostriata*.

### 3.2. StaHemF5 May Be Involved in the Regulation of Leaf Color Formation in S. tootsik f. albostriata

HemF encodes coproporphyrinogen III oxidase (CPOX), which catalyzes a key enzymatic step at the branch point of the tetrapyrrole biosynthetic pathway, leading to the production of chlorophyll and heme [8,21]. Dysfunction of CPOX has been repeatedly associated with impaired chlorophyll accumulation, defective chloroplast development, and abnormal leaf coloration in higher plants [22,23]. Among the six *StaHemF* family members identified in this study, *StaHemF5* was selected as the most promising candidate based on integrated evolutionary and expression analyses. Phylogenetic analysis showed that *StaHemF5* clustered with functionally characterized CPOX homologs from Poaceae species and contained conserved structural domains associated with CPOX function. Furthermore, subcellular localization prediction and transient expression analysis in *N. benthamiana* confirmed that *StaHemF5* is localized in chloroplasts, supporting its potential involvement in chlorophyll biosynthesis and tetrapyrrole metabolism.

Expression analysis further revealed that *StaHemF5* transcript levels were significantly higher in WW than in WG leaf buds at the S3 stage based on qRT-PCR results. However, RNA-seq-derived FPKM values showed an opposite expression pattern at this stage, indicating a discrepancy between transcriptome quantification and qRT-PCR validation. Such inconsistencies may arise from several technical factors, particularly in species with multiple homologous gene members. During de novo transcript assembly and abundance estimation, highly conserved sequences among gene family members may result in ambiguous read assignment and affect transcript abundance calculations, whereas qRT-PCR provides higher specificity when gene-specific primers are used [24,25,26]. Although the present results demonstrate a close association between *StaHemF5* expression and leaf color variation, and transient overexpression in tobacco further supports its positive regulatory effect on chlorophyll accumulation, the direct contribution of *StaHemF5* to the albino phenotype of *S. tootsik* f. *albostriata* requires further verification. Future functional studies using loss-of-function approaches, such as CRISPR/Cas9-mediated gene editing or RNA interference, will be necessary to determine whether *StaHemF5* directly controls chlorophyll deficiency or whether its increased expression in WW leaf buds represents a compensatory response triggered by impaired chlorophyll biosynthesis. These studies will provide further insights into the regulatory network underlying leaf color formation in ornamental bamboo.

### 3.3. StaHemF5 Influences Leaf Color Formation by Regulating CPOX Activity and Chlorophyll Accumulation

Transient overexpression of *StaHemF5* in tobacco resulted in a darker green leaf phenotype accompanied by significant increases in chlorophyll content and CPOX enzyme activity. As CPOX catalyzes the conversion of coproporphyrinogen III to protoporphyrinogen IX, enhanced *StaHemF5* activity is expected to promote metabolic flux through the tetrapyrrole pathway and increase precursor availability for chlorophyll biosynthesis. These findings provide functional evidence that *StaHemF5* acts as a positive regulator of chlorophyll accumulation, consistent with the reported role of *FsHemF* in Forsythia [22]. However, in WW leaf buds of *S. tootsik* f. *albostriata*, increased *StaHemF5* transcript abundance did not result in elevated CPOX activity or chlorophyll accumulation, indicating that transcriptional activation alone is insufficient to restore pigment production in the albino background. This apparent discrepancy likely reflects the complex regulation of chlorophyll biosynthesis, which depends not only on gene expression but also on plastid developmental status, substrate availability, protein stability, and metabolic feedback mechanisms [26,27,28,29].

Previous studies have shown that severe impairment of chloroplast structure can disrupt coordination between nuclear and plastid genomes and compromise the function of nuclear-encoded chloroplast proteins [30,31,32]. Considering that WW leaf buds exhibit abnormal chloroplast development, the elevated expression of *StaHemF5* may represent a compensatory response to chlorophyll deficiency rather than an effective mechanism for restoring pigment accumulation. These findings suggest that *StaHemF5* requires an intact chloroplast metabolic environment to fully exert its regulatory function. Further investigation of CPOX protein abundance, enzyme activity regulation, and tetrapyrrole intermediate accumulation will be necessary to elucidate the mechanism underlying the uncoupling between *StaHemF5* transcription and chlorophyll accumulation in the albino phenotype. Such studies will help clarify how chlorophyll biosynthetic genes interact with plastid development to determine leaf color formation in *S. tootsik* f. *albostriata*.

## 4. Materials and Methods

### 4.1. Plant Materials and Growth Conditions

One-year-old plants of *Sinobambusa tootsik* f. *albostriata*, propagated through division, were cultivated in the nursery of the Bamboo Research Institute at Fujian Agriculture and Forestry University under 50% shading provided by shade nets. The photosynthetic photon flux density (PPFD) was approximately 600–800 μmol·mm^−2^ s^−1^ during cultivation. *Nicotiana benthamiana* plants were grown in a climate-controlled chamber under a PPFD of 150 μmol with a 16 h light/8 h dark photoperiod, 22–25 °C temperature, and 60–70% relative humidity.

### 4.2. RNA Extraction and Transcriptome Sequencing Analysis

**Figure 12:**
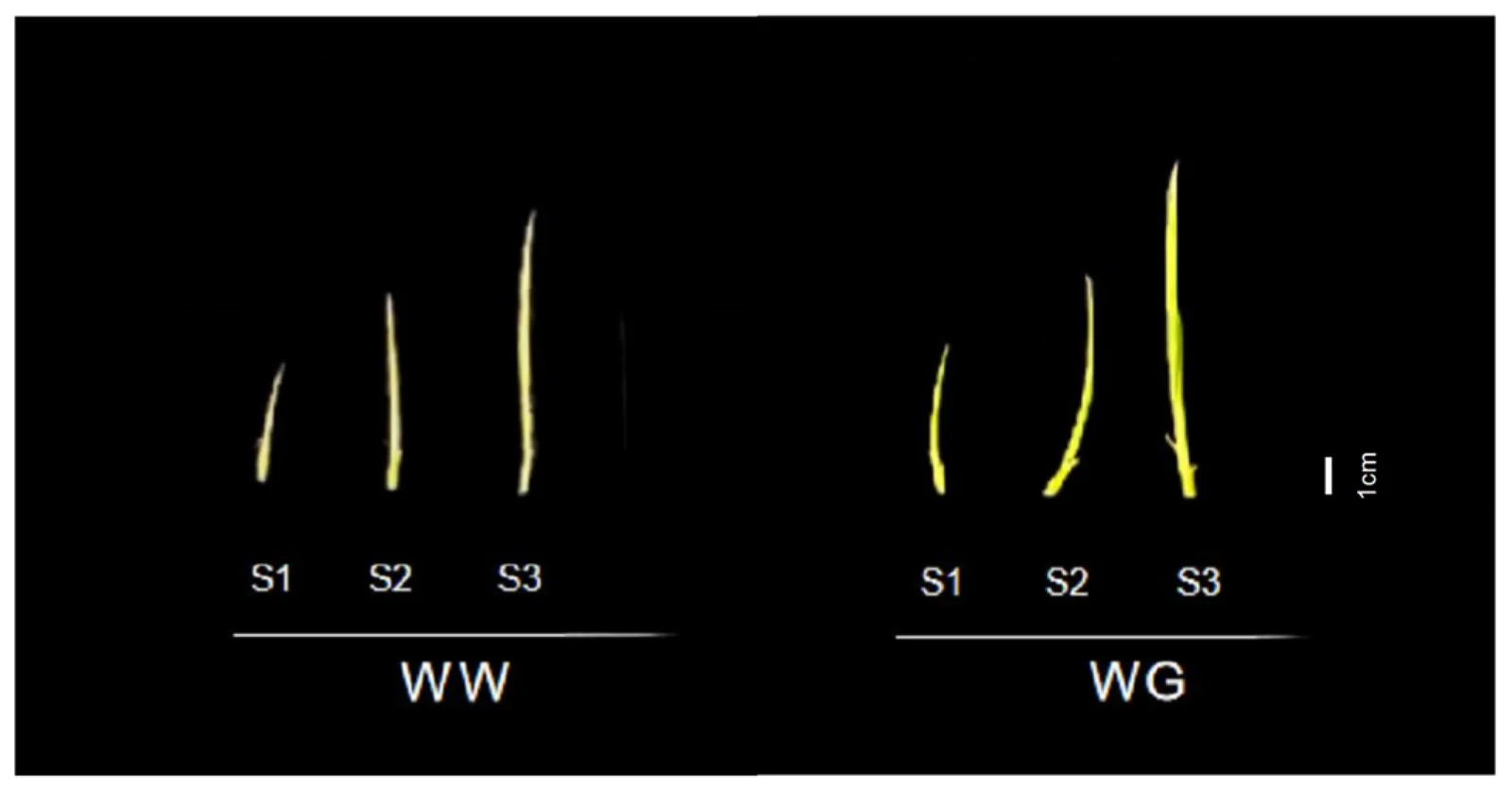
Sample of *S. tootsik* f. *albostriata* test material.

Leaf buds of *S. tootsik* f. *albostriata* exhibiting the WW and WG phenotypes were collected at three developmental stages according to bud length, including the S1 stage (1–3 cm), S2 stage (3–6 cm), and S3 stage (6–9 cm) (Figure 12). Three independent biological replicates were prepared for each developmental stage. The collected samples were immediately frozen in liquid nitrogen and stored at −80 °C until further analysis. All samples were subsequently submitted to Wuhan Metware Biotechnology Co., Ltd. (Wuhan, China) for transcriptome sequencing and subsequent data processing.

The transcriptome datasets used in this study were obtained from previously completed RNA-seq experiments of *S. tootsik* f. *albostriata*. Among the available developmental stages and leaf color phenotypes, three developmental stages (S1–S3) and two representative phenotypes, green–white (WG) and white–white (WW), were selected for transcriptome analysis. These samples were selected because S1–S3 represent critical developmental stages associated with leaf color differentiation, and the comparison between WG and WW provides a clear contrast between green and white tissues. The transcriptome data were analyzed to identify differentially expressed genes potentially involved in chlorophyll metabolism and leaf color formation.

RNA concentration and integrity were assessed prior to library construction. Polyadenylated mRNA was enriched using oligo (dT) magnetic beads, followed by RNA fragmentation, double-stranded cDNA synthesis, end repair, adapter ligation, fragment size selection, and PCR amplification to construct cDNA libraries. After quality assessment, qualified libraries were subjected to high-throughput sequencing on the Illumina platform [33]. The raw sequencing reads were filtered through quality control procedures to obtain clean reads. Since no reference genome was available, de novo transcriptome assembly was performed using Trinity (version 2.15.1), and unigene sequences were generated by clustering transcripts with Corset (version 1.09) [34]. For functional annotation, the assembled unigenes were aligned against seven public databases using DIAMOND BLASTX (version 2.1.8) and HMMER (version 3.3.2) software [35,36,37]. Differentially expressed gene (DEG) analysis was performed using DESeq2 (v1.38.3) and edgeR (version 3.40.2) for datasets with and without biological replicates, respectively [38,39]. The Benjamini–Hochberg procedure was applied for multiple testing correction. Genes with |log2(fold change)| ≥ 1 and false discovery rate (FDR) < 0.05 were considered significant DEGs and were subsequently subjected to functional enrichment analysis [40,41].

### 4.3. Determination of Chlorophyll Content

Chlorophyll content was determined in WW and WG leaf samples of *S. tootsik* f. *albostriata* collected at three developmental stages (S1–S3). Briefly, 0.1 g of fresh tissue was homogenized and extracted in 5 mL of anhydrous ethanol in darkness for 24 h to ensure complete pigment extraction. The absorbance of the extracts was measured at 665 nm and 649 nm using a spectrophotometer (Thermo Fisher Scientific, Waltham, MA, USA), with three technical replicates for each sample. The contents of chlorophyll a (C_a_), chlorophyll b (C_b_), and total chlorophyll (C_t_) were calculated according to the following equations [42]:

C_a_ (mg·L^−1^) = 13.95A_665_ − 6.88A_649_

C_b_ (mg·L^−1^) = 24.96A_649_ − 7.32A_665_

C_t_ (mg·L^−1^) = C_a_ + C_b_

Pigment content (mg·g^−1^ FW) = (C × V)/(1000 × W)

where C_a_ and C_b_ represent the concentrations of chlorophyll a and chlorophyll b, respectively (mg·L^−1^); C_t_ represents the total chlorophyll concentration (mg·L^−1^); C represents the pigment concentration calculated from the above equations (mg·L^−1^); V represents the total volume of extraction solvent (mL); and W represents the fresh weight of the leaf bud sample (g).

### 4.4. CPOX Enzyme Activity Assay

CPOX enzyme activity was determined using a plant coproporphyrinogen III oxidase (CPOX) ELISA kit (Fuzhou Qingbaiwang Biotechnology Co., Ltd., Fuzhou, China) according to the manufacturer’s instructions. Briefly, standards and sample extracts were added to CPOX antibody-coated microplates, followed by the addition of an HRP-conjugated detection antibody. The plates were incubated at 37 °C for 60 min and washed five times to remove unbound components. Subsequently, substrate solutions A and B were added, and the reaction was incubated at 37 °C in the dark for 15 min. After the addition of the stop solution, absorbance was measured at 450 nm using a microplate reader. A standard curve was generated using Microsoft Excel, and CPOX enzyme activity in each sample was calculated by interpolation from the standard curve and corrected according to the dilution factor. The enzyme activity was expressed as international units per gram of fresh weight (IU·g^−1^ FW).

### 4.5. Screening of StaHemF Candidate Genes in S. tootsik f. albostriata

Based on transcriptome functional annotation results, candidate *StaHemF* genes were screened according to the criteria of |log2(fold change)| > 1 and adjusted *p*-value (padj) < 0.05. The identified candidates were further validated through homologous alignment with HEMF protein sequences from multiple plant species. HEMF protein sequences from diverse terrestrial plants and selected outgroup species were obtained from three publicly available plant genome databases, including TAIR (https://www.arabidopsis.org/), RAP-DB (https://rapdb.dna.affrc.go.jp/, accessed on 17 June 2026), and EnsemblPlants (https://plants.ensembl.org/index.html, accessed on 17 June 2026). The selected species included dicotyledonous model plants, such as *Arabidopsis thaliana*, *Nicotiana tabacum*, *Solanum lycopersicum*; monocotyledonous grass species, including *Oryza sativa*, *Zea mays*, *Brachypodium distachyon*, *Setaria italica*, *Sorghum bicolor*, and *Triticum aestivum*; bamboo species, including *Phyllostachys edulis* and *Dendrocalamus latiflorus*; and outgroup species, including the moss *Physcomitrium patens* and Chlamydomonas reinhardtii. A phylogenetic tree was constructed using MEGA (version 11.0) software with the Neighbor-Joining method, 1000 bootstrap replicates, and the Poisson model [43]. Expression clustering heatmaps were generated using TBtools (version 2.458) with hierarchical clustering based on Euclidean distance [44].

### 4.6. Bioinformatics Analysis of StaHemF Protein

The complete open reading frame (ORF) and deduced amino acid sequence of *StaHemF* were identified using the ORF Finder tool available from the NCBI database (https://www.ncbi.nlm.nih.gov/orffinder/, accessed on 7 August 2026) [45]. The physicochemical properties of the StaHemF protein, including molecular weight, theoretical isoelectric point, instability index, and hydrophilicity, were analyzed using the ExPASy ProtParam tool. Physicochemical properties of the protein, including relative molecular mass, instability index, and hydrophilicity, were analyzed on the ExPASy-ProtParam platform (https://web.expasy.org/protparam, accessed on 7 August 2026) [46]. Transmembrane regions were predicted using TMHMM-2.0 (https://services.healthtech.dtu.dk/services/TMHMM-2.0/, accessed on 7 August 2026) [47], and signal peptide prediction was performed using SignalP 6.0 (https://services.healthtech.dtu.dk/service.php?SignalP-6.0, accessed on 7 August 2026). Potential phosphorylation sites were predicted using the NetPhos 3.1 server (https://services.healthtech.dtu.dk/services/NetPhos-3.1/, accessed on 7 August 2026) [48]. The secondary structure and three-dimensional structure of *StaHemF* were predicted using the SOPMA online tool (https://npsa-pbil.ibcp.fr/cgi-bin/npsa_automat.pl?page=/NPSA/npsa_sopma_f.html, accessed on 7 August 2026) and the SWISS-MODEL server (http://swissmodel.expasy.org, accessed on 7 August 2026), respectively. Subcellular localization was predicted using WoLF PSORT (https://wolfpsort.hgc.jp/, accessed on 7 August 2026) [49,50]. All analyses were performed using the default parameters of each online tool. Multiple sequence alignment and homology analysis were conducted using DNAMAN software (version 9).

### 4.7. Expression Specificity Analysis of StaHemF

Specific primers were designed using the NCBI Primer-BLAST tool (https://www.ncbi.nlm.nih.gov/tools/primer-blast/, accessed on 7 August 2026), and the primer sequences are listed in Table S2. Total RNA was extracted from WW and WG leaf buds of *S. tootsik* f. *albostriata* at three developmental stages (S1, S2, and S3) using an RNA extraction kit (Vazyme Biotech Co., Ltd., Nanjing, China). First-strand cDNA was synthesized using the PrimeScript™ II 1st Strand cDNA Synthesis Kit (Takara Biotechnology (Dalian) Co., Ltd., Dalian, China) according to the manufacturer’s instructions.

Quantitative real-time PCR (qRT-PCR) was performed on an Applied Biosystems QuantStudio 3 Real-Time PCR System (Thermo Fisher Scientific, Waltham, MA, USA), using Hieff® qPCR SYBR Green Master Mix (Low Rox Plus) (Yeasen Biotechnology Co., Ltd., Shanghai, China). Each 20 μL reaction mixture contained 10 μL of SYBR Green Master Mix, 0.4 μL of each forward and reverse primer, 2 μL of cDNA template, and RNase-free water to the final volume. The amplification program consisted of an initial denaturation at 95 °C for 5 min, followed by 40 cycles of 95 °C for 10 s and 60 °C for 30 s. A melting curve analysis was subsequently performed using the default settings of the instrument to verify amplification specificity. Each sample was analyzed using three independent biological replicates and three technical replicates. The EF1α gene was used as the internal reference gene, and relative gene expression levels were calculated using the 2^−ΔΔCt^ method [51].

### 4.8. Cloning of StaHemF Gene from S. tootsik f. albostriata

The open reading frame (ORF) of *StaHemF* was predicted using the NCBI ORF Finder tool. Nested PCR primers were designed using Primer Premier5 (version 5.00) and SnapGene software (version 6.0.2), and the primer sequences are listed in Table S2. Total RNA was extracted according to the method described in Section 4.7. First-strand cDNA was synthesized according to the method described in Section 4.7. Nested PCR amplification was conducted using a GET3XG Thermal Cycler (Hangzhou Bioer Technology Co., Ltd., Hangzhou, China). The amplified products were purified using the UElandy® BIOKIT UE DNA Gel Extraction Kit (UElandy Biotechnology Co., Ltd., Shanghai, China).

### 4.9. Subcellular Localization Analysis of StaHemF5 Protein

The recombinant vector pCAMBIA1302-*StaHemF*-GFP and the empty vector pCAMBIA1302 (Tsingke Biotechnology Co., Ltd., Beijing, China) were separately introduced into *Agrobacterium tumefaciens* strain GV3101 (Tsingke Biotechnology Co., Ltd., Beijing, China). After incubation and colony PCR confirmation, positive bacterial cultures were diluted 50-fold and resuspended in infiltration buffer containing 10 mM MgCl_2_ (Takara Biotechnology (Dalian) Co., Ltd., Dalian, China), 10 mM MES (pH 5.6) (Takara Biotechnology (Dalian) Co., Ltd., Dalian, China), and 150 uM acetosyringone (Takara Biotechnology (Dalian) Co., Ltd., Dalian, China). The bacterial suspension was adjusted to an OD600 of 1.0 and infiltrated into the abaxial surface of leaves from four-week-old *Nicotiana benthamiana* plants using a needleless syringe (Takara Biotechnology (Dalian) Co., Ltd., Dalian, China). After incubation for 60–72 h under normal growth conditions, infiltrated leaf areas (0.5–1 cm^2^) were excised and examined using a laser scanning confocal microscope (LSM 880, Carl Zeiss, Oberkochen, Germany) [52].

### 4.10. Verification of Transient Overexpression Function in Tobacco

The recombinant vector pCAMBIA1301::*StaHemF* and the empty vector pCAMBIA1301 (Tsingke Biotechnology Co., Ltd., Beijing, China) were separately introduced into *Agrobacterium tumefaciens* strain GV3101. The resulting overexpression strains were cultured, and infiltration suspensions were prepared according to the procedures described in Section 4.9. Following infiltration, *Nicotiana benthamiana* plants were maintained in a controlled growth chamber at 22–25 °C under a 16 h light/8 h dark photoperiod. Leaf samples were collected at different time points after infiltration for subsequent analyses. Specifically, samples were harvested at 72 h post-infiltration for qRT-PCR analysis, at 96 h post-infiltration for CPOX activity determination (Section 4.4), and at 6–7 days post-infiltration for chlorophyll content measurement (Section 4.3), based on the established transient expression kinetics. The primers used in this study are listed in Table S2.

### 4.11. Statistical Analysis

Statistical analysis of significant differences was carried out using IBM SPSS software (version 27.0.1.0). Graphic representations were generated using OriginPro 2024 (version 10.1.0.178 ) and GraphPad Prism (version 11.0.0)

## 5. Conclusions

This study systematically investigated the molecular characteristics and biological function of *StaHemF*, a gene associated with chlorophyll accumulation, in *Sinobambusa tootsik* f. *albostriata*. By integrating physiological measurements, transcriptomic analysis, bioinformatic characterization, and functional validation, we demonstrated that chlorophyll accumulation is closely associated with green–white leaf color differentiation, with early leaf bud development representing a critical period for phenotypic establishment. Among the six identified *StaHemF* family members, *StaHemF5* was selected as the most promising candidate based on its evolutionary conservation and distinct expression characteristics. *StaHemF5* encodes a chloroplast-localized protein that exhibits high conservation among grasses and bamboo species, supporting its potential conserved function in chlorophyll biosynthesis. Functional validation through transient overexpression in *Nicotiana benthamiana* showed that *StaHemF5* enhanced CPOX activity and promoted chlorophyll accumulation, indicating its positive regulatory role in chlorophyll metabolism. Collectively, these findings identify *StaHemF5* as an important candidate gene associated with leaf color formation in *S. tootsik* f. *albostriata* and provide new insights into the molecular mechanisms underlying bamboo leaf color variation. This study establishes a foundation for further elucidation of the regulatory network governing chlorophyll metabolism and provides a valuable genetic resource for molecular breeding and improvement of ornamental bamboo germplasm.

## Figures and Tables

**Table 1:** Genetic ID numbers and names.

Gene	ID	Full-Length Gene/bp	CDS/bp
*StaHemF1*	Cluster-60390.5	1762	396
*StaHemF2*	Cluster-60390.3	1928	519
*StaHemF3*	Cluster-44301.0	1053	1050
*StaHemF4*	Cluster-60390.1	2792	372
*StaHemF5*	Cluster-60390.0	2145	1176
*StaHemF6*	Cluster-60390.7	423	333

**Table 2:** Physicochemical properties of amino acid sequences of StaHemF genes.

Protein	Mr(Da)	PI	Formula	Arg + Lys	Asp + Glu	Instability Index	GRAVY
StaHemF5	43,501.02	6.48	C_1953_H_2976_N_544_O_569_S_10_	49	51	61.06	−0.472

**Table 3:** Types and contents of amino acids in StaHemF genes.

Protein	Ala	Arg	Asn	Asp	Cys	Gln	Glu	Gly	His	Ile
StaHemF5	9.5%	7.7%	2.0%	5.9%	1.5%	3.1%	7.2%	9.2%	2.0%	3.8%
**Protein**	**Leu**	**Lys**	**Met**	**Phe**	**Pro**	**Ser**	**Thr**	**Trp**	**Tyr**	**Val**
StaHemF5	6.4%	4.9%	1.0%	5.9%	7.7%	6.1%	4.6%	2.0%	3.3%	6.1%

**Table 4:** Subcellular localization prediction results of StaHemF protein.

Subcellular Localization Sites	Predicted Score
Chloroplast (chlo)	13
Cytosol-mitochondrion (cyto_mito)	1

## Data Availability

The processed RNA-seq datasets analyzed in this study, including gene expression matrices and differential expression analysis results, have been provided in the Supplementary Materials. Additional datasets generated during this study are available from the corresponding author upon reasonable request.

## Supplementary Materials

The following supporting information can be downloaded at: https://www.mdpi.com/article/10.3390/ijms27167186/s1.
