# Supplementary material for: Screening and Functional Validation of the Chlorophyll Synthesis-Related Gene StaHemF in Sinobambusa tootsik f. albostriata
Source: Int J Mol Sci. 2026 Aug 11;27(16):7186. doi: 10.3390/ijms27167186 (PMC13513669; doi:10.3390/ijms27167186)
Supplement: Supplementary file 1 [file ijms-27-07186-s001.zip › Supplementary Figure S1.pdf]

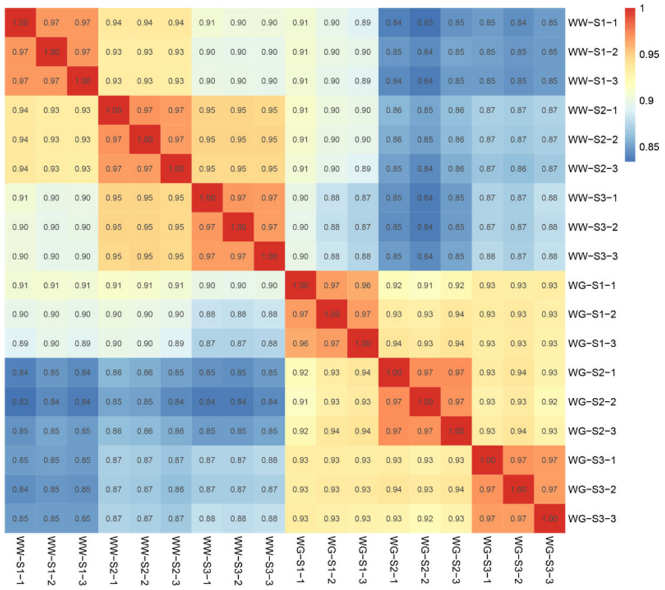

Supplementary Figure S1: Pearson correlation heatmap of transcriptome samples of *Sinobambusa tootsik* f. *albostriata*. Pearson correlation coefficients among all samples were calculated based on gene expression levels to assess the reliability and reproducibility of transcriptome sequencing data.
